# Barriers Towards Obstetric Care Service Utilization in Ethiopia: An Explorative Qualitative Study

**DOI:** 10.4314/ejhs.v33i2.4S

**Published:** 2023-10

**Authors:** Alemu Tamiso, Meskerem Jisso, Netsanet Abera, Akalewold Alemayehu, Anteneh Gadisa, Abdurezak Umer, Mesfin Kebede, Hussen Mohammed, Bekele Yazie, Habtamu Sime Gizaw, Biru Abdissa Mizana, Elias Ali Yesuf, Binyam Tilahun, Berhanu Fikadie Endehabtu, Tajebew Zayede Gonete, Kassahun Dessie Gashu, Dessie Abebew Angaw, Kassu Ketema Gurmu, Rekiku Fikre

**Affiliations:** 1 Hawassa University, College of Medicine and Health Sciences, Ethiopia; 2 Dire Dawa University, College of Medicine and Health Sciences, Ethiopia; 3 Jimma University, Institute of Health, Ethiopia; 4 University of Gonder, College of Medicine and Health Science, Institute of Public Health, Ethiopia; 5 World Health Organization Country Office for Ethiopia, Universal Health Coverage/Life Course, Health System Strengthening Team, Addis Ababa, Ethiopi

**Keywords:** obstetrics care, barrier, Ethiopia

## Abstract

**Background:**

Obstetric care has been at the center of both global and national agendas. More than 50% of pregnant mothers are still preferring to give birth at home with some even after having full antenatal care. However, a few literatures looked at contributing factors for this problem but they are not conclusive and do not consider different sociocultural context of Ethiopia and different health service related barriers. Hence, the aim of this study was to explore barriers to obstetric care service utilization in Ethiopia using the socio-ecological model.

**Methods:**

Explorative qualitative study was employed involving key-informant interviews, in-depth interviews, and focus group discussions between October and December 2021; Individual, community, health system, and contextual barriers were explored. Atlas ti. Version 9 was used for analysis.

**Result:**

Lack of awareness, unfavorable perception, lack of partner involvement, cultural barrier, shortage of supplies, poor infrastructure, provider-related factors, poor monitoring, and evaluation system, challenging topography, and conflict were the major barriers that hinder mothers from receiving obstetrics service in Ethiopia.

**Conclusion:**

Lack of awareness, unfavorable perception, conflict, problems with health system structure and process, and cultural and geographical conditions were major barriers in Ethiopia. Therefore, packages of intervention is important to avail essential equipment, strengthening follow up system, create awareness, and increase access to health facilities is very important for service improvement by the government and non-governmental organizations. Additionally, implementing conflict resolution mechanism is important for addressing better obstetric service.

## Introduction

Obstetric care encompasses the provision of care to pregnant women throughout the stages of pregnancy, labor, and the postpartum period. This care is delivered by skilled health professionals who have received appropriate training and can be accessed at various healthcare facilities (1). Its primary objectives include identifying and managing risks, preventing diseases, and promptly addressing any danger signs. Additionally, obstetric care offers pregnant women the opportunity to acquire knowledge about healthy behaviors, reduces the risk of mother-to-child HIV transmission, and provides access to services during and after pregnancy (2).

Skilled birth attendance, a crucial component of obstetric care, has demonstrated its ability to significantly reduce maternal deaths, accounting for more than three-fourths of the decline. This intervention is widely regarded as the most critical measure for reducing maternal mortality (3). Nevertheless, the availability, effectiveness, and utilization of obstetric care in developing countries are influenced by a multitude of factors, both health-related and non-health-related. These factors contribute to the occurrence of preventable maternal and newborn deaths. According to the World Health Organization, approximately 810 per 100,000 women in low- and middle-income countries (LMICs) die every day due to avoidable causes associated with pregnancy and childbirth (4). Consequently, each stage of obstetric care plays a pivotal role in mitigating the unacceptably high rates of maternal and newborn mortality in LMICs.

Sexual and reproductive health and rights in general, and obstetric care specifically, have been at the center of both global and national agendas. There were several global initiatives and goals set to improve obstetric care such as safe motherhood, the millennium development goal, and the recently sustainable development goal (SDG); regrettably, only five countries achieved the millennium developmental goal (MDG5) (improve maternal health) with wide geographic inequities in overall performance (5). The SDG in its goal three aims to ensure healthy lives and promote well-being for all people of all ages. However, the recent global pandemic has already hampered efforts, forcing us to assess possibilities with assumptions such as the SDG push scenario, COVID baseline scenario, and high damage scenario, each showing less probability of meeting the goal (6). In Ethiopia, reproductive health with a greater focus on obstetric care has been on the agenda for the last two decades (7)(8); it is worth noting that a policy analysis reported that the nation's maternal and child health policies failed to elucidate plans to implement and monitor the proposed interventions and ended with a strong suggestion to focus on equity (9).

Between 2000 and 2016, Ethiopia saw a significant drop in maternal mortality, going from 871 deaths per 100,000 live births to 412 deaths per 100,000 live births, but still far higher than the global average (10,11)(12). More than 50% of pregnant mothers are still preferring to give birth at home with some even after having full antenatal care citing reasons such as poor quality of care, cultural reasons, and disrespectful and abusive care among others (13–16). However, few looked at demand-side barriers and almost none looked at the national level. Hence, this study attempted to explore the demand side view of barriers to the utilization of obstetrics care in Ethiopia.

## Materials and Methods

**Study setting and design:** The study was undertaken in four regions of Ethiopia namely Amhara, Oromia, Southern Nations and Nationalities of Peoples' (SNNP), and Sidama regions between October and December 2021. To address the objective of this study an explorative qualitative study was employed by involving key-informant interviews, in-depth interviews, and focus group discussions.

**Participants:** Maternal and child health (MCH) case team leaders, health extension workers (HEWs), regional, zonal, and Woreda health office (WoHO) level reproductive maternal and nutrition and child health (RMNCH) experts were participants for key informant interviews (KII). Pregnant mothers and home delivered mothers were participants for the study in-depth interview (IDI). Focus group discussions (FGDs) were conducted with community leaders (women's development team (WDT), religious and kebele leaders, traditional healers, and traditional birth attendants), and hospital quality leaders or members.

**Sample size and sampling:** Study participants were selected in consultation with regional health bureaus, respective WoHO and HEWs. For the key-informant and in-depth interview, participants were selected using a maximum variation sampling. In maximum variation sampling, participants' selection is often made using pre-set criteria to ensure the inclusion of as many variant observations as possible. These variations can result from variations in the demography of the participants or the phenomenon. In this study, maximum variations were achieved by including participants from different geographical locations (it was conducted in different regions of Ethiopia), age groups (from adolescent to elderlies), gender, and role in the community and health facility. FGD participants were purposively selected in the community. The sample size was determined by the level of saturation of the collected information.

A total of 41 participants (regional RMNCH directors, woreda RMNCH coordinators, Primary Health care unit (PHCU), and MCH case team leaders) participated in the KIIs, whereas 32 people participated in the IDIs (15, 5, 6 and 6 at SNNP, Sidama, Oromia and Amhara regions, respectively). A total of 13 FGDs (4 at SNNP, 3 FGD at each Sidama, Oromia and Amhara regions) were undertaken considering agrarian, rural, and urban places and male to female equal participation were also considered. Because of the cultural sensitivity of the SRMH services, clients who participated in IDI did not participate in the FGD.

**Data collection Tool and procedure:** Pre-tested key-informant, in-depth interview, and focus group discussion guides were used for collecting relevant data. The tools were developed to identify barriers of obstetric care service utilization in Ethiopia. The interview guides were categorized into four main themes of the socio-ecological model; namely, individual, community, health system, and contextual level barriers and different questions related to the sub-themes were asked with different probing mechanisms.

In consultation with regional health bureaus 19 woredas from 5 zones of SNNP region, 9 woredas from Sidama region, 10 woredas from 4 zones of Amhara region and 8 woredas from 2 zones of Oromia region were selected purposively. Health facilities were also selected by WoHO. Community leaders and IDI participants were also selected purposively in collaboration or consultation with HEWs and respective WoHO. During the selection process different age category, from adolescent to elderly, and equality of male to female distribution were considered.

All woredas were classified in to three sections, high performing, medium performing and low performing then all KIIs, IDIs and FGDs were conducted proportionally.

**Data Quality Assurance:** Training had been provided to data collectors and supervisors about the purpose and content of the tools before the data collection. A pre-test was undertaken in woredas of all regions' other than the actual data collection area before the actual data collection started. Close follow-up by the research team and supervisors was undertaken, and feedback was also given on the completed forms for the data collectors before the next data collection day. A quality check was done after transcription to check the consistency of the transcription with the recorded audio and way of writing. The quality of the verbatim transcriptions and translations was assessed by a team of experts.

**Data management and analysis:** For analysis, the data were transcribed and then translated into English. To clarify issues, field notes were added to the transcripts. Researchers read the transcripts before beginning coding and writing them up. The pre-defined codes were used to get a complete picture of the data. Atlas ti. version 9 was used to evaluate the qualitative data using a thematic content analysis approach using a socio-ecological model.

**Ethical consideration:** Ethical clearance was obtained from the Institutional Review Board of Hawassa University, College of Medicine and Health Sciences. An official permission letter was obtained from the regional health offices, WoHO, and health facilities, respectively. Official permission was also sought from the selected health facilities. After confirmation to participate in the study, written informed consent was obtained from participants. Personal privacy was maintained by interviewing the interviewee alone and identifications like names were not used in the questionnaire. Participants were also assured that their participation, nonparticipation, or refusal to answer questions did not affect their personal lives.

## Results

**Socio-demographic characteristics:** The participants' age ranged from 19 to 68, and their level of education ranged from being able to read and write up to have a degree (table 1).

**Table 1:** socio-demographic characteristics of participants for barriers to obstetrics health service utilization in Ethiopia, 2021

Socio-demographic characteristics	KII and IDI (n=73)	Focus group discussion (n=13)
Age		
19-29	12 (16.4)	14(14.6)
30-40	31(42.5)	30 (31.3)
41-50	19(26.0)	33(34.4)
51+	11 (15.1)	19(19.8)
Sex		
Male	49(67.1)	47 (48.9)
Female	24(32.9)	49(51.1)
Occupation		
Health worker	45(61.6)	59(61.5)
Community leader		37(38.5)
Pregnant women	12 (16.4)	
House wife	16(21.9)	

Note: KII: Key informant interview, IDI: In depth Interview

**Fig. 1:**
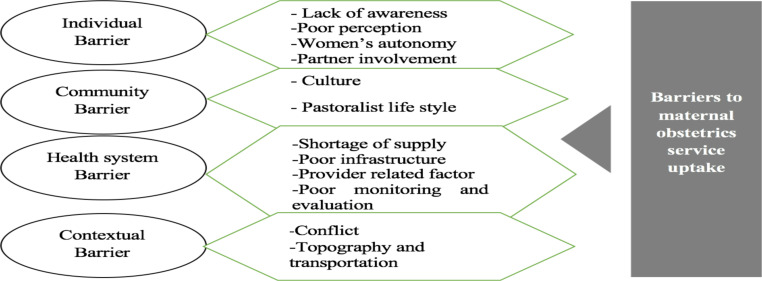
Socio-ecological model that become barriers in utilizing Maternal obstetrics services in Ethiopia, 2021

**Barriers to maternal obstetrical service utilization:** Utilizing a socio-ecological model, barriers to women's obstetrics service uptake were compiled into four main themes: individual, community, health system, and contextual level (Fig. 1).

### 1. Individual level barriers

Maternal obstetric service utilization is mainly affected by lack of awareness, religion, unfavorable perception, and socio-cultural barriers.

**Lack of knowledge:** Most FGD and KII participants mentioned that lack of awareness about when and where to get and start obstetric services and the effect of significant others were the main causes of late initiation of antenatal care (ANC) services and home delivery.

*“It is due to low awareness; they refer to the previous ancestors not using the service. They said that our mothers didn't attend any antenatal care services during their pregnancies and gave normal births. So why do we go to a health facility unless we are diseased? Especially women with a mother-in-law nearby, don't attend it because they are influenced by them not to attend.”* [48-year-old FGD participant, WDT leader, Amhara region]

Similarly, one IDI participant mentioned that mothers do not come for delivery services because of their religious perceptions.

*“They claim that whether we visit a medical facility or not, Allah will protect us from birth complications and give us a healthy baby.”* [29 years mother who gave birth at home, Oromia region]

The barrier to early initiation of ANC was further explained by a respondent from the SNNP region who indicated a lack of awareness and preference for late initiation were major conditions.

*“I don't think we have a problem from their side except that they don't have awareness about when to start the service. Those who have know-how also prefer to initiate the ANC when the pregnancy gets physically visible. So, it is more about awareness and preference to come late”* [30-year-old, KII, SNNPR, WrHO MCH focal]

**Unfavorable perception and workload:** The majority of the participants stated that mothers seek obstetrics care if they are sick, which is the main reason for the high ANC default rate and low PNC service utilization. This finding was described by a key informant participant from the Southern region and reported as follows;

*“Others don't think that ANC is needed for a healthy mother. If they don't feel any health problems, they don't want to come after they attend the first visit. Some mothers come for delivery service only after they have their first ANC visit.”* [31 years old female KII participant, MCH focal, SNNPR].

Most IDI participants also mentioned that high workloads in their homes and unfavorable attitude toward PNC service uptake were barriers.

*“Everyone thinks of their home activity. No one considers PNC as important as other services like ANC and delivery services. No one is interested in having PNC services; they were highly eager to go to their home once they gave birth. In-home there are animals like cattle, hens,… which need women's attention. Most of the women and the other community didn't want to go to health facilities unless they suffered painful conditions. They believed there would be no problem at all after a safe delivery.”* [26 year old home deliver mother, Amhara region].

In addition, other participants also further described the need for confidentiality and traditional practice of mothers selecting home delivery.

*“Mothers like to get heat, so they like to be covered by plenty of clothes because they think it helps for her to deliver. That is why they hate health centers.”* [KII, Sidama region, WrHO MCH coordinator].

#### Partner involvement

Most participants indicated that their husbands' involvement was an important factor in the utilization of obstetrics services.

“…*Involving husbands increases utilization of ANC, delivery, and PNC, but we are not involving them. This could be the other reason for the low utilization of maternal obstetrics health services.”* [47-year-old KII participant, RMCH officer, Amhara region]

Conversely, a significant number of interviews from the Southern and Sidama regions mentioned that husbands do not usually oppose ANC use but prefer not to go together.

*“Husbands are positive for service utilization because the kebele structures are also enabling the father to accompany their wife. However, they don't want to come together with her.” [34-year-old* KII participant, RMNCH Officer, SNNPR]

### 2. Community level barriers

**Culture:** Most of the participants explained how cultural beliefs hinder them from utilizing the health services because of how they relate early pregnancy to possible evil attacks as one of the obstacles to utilizing the ANC service.

*“An obstacle for ANC service is that pregnant women don't want to show their pregnancy to others. They cover their womb with clothes until it becomes bigger. They do not even want to tell their husband either. There is a fear of evil spirits and the belief that they shouldn't tell anybody till the pregnancy is exposed by itself. They come after about a 7^th^ month. She could deliver before receiving adequate ANC services.”* [36 years KII participant, RMNCH director, SNNPR]

The majority of participants consistently also reported that cultural beliefs were their major barrier to low PNC service utilization. They think evil attacks the postpartum mother and baby if the mother leaves the house before 40 days of delivery. A participant from southern Ethiopia said that:

*“… mother came to our health facility on their forty-fifth day for vaccination program not for PNC service, because there is a general custom that prevents them from going out of the house after delivery. They believe that if they leave their house before 40 days they would expose to cold and evil attacks. They also say ‘nifas yemetal’ if they leave their home early. After all, no mother comes for PNC rather we go to their homes.”* [KII, SNNPR, PHCU MCH focal]

Another participant from the pastoralist area reaffirmed how much cultural belief affects the utilization of obstetrics services, specifically delivery services.

*“… our culture prevents the mother from having a health facility delivery. They give birth in the jungle alone and return to their home after three days.”* [44 years old, KII participant, Zonal MCH coordinator, SNNPR]

The pastoralist lifestyle is also another barrier for receiving obstetrics services appropriately, and it was further explained by a key informant from the pastoralist area:

*“… Some are coming in the 6^th^ or 7^th^ month. Others move to wetlands with their cattle. They either use it there or leave it at all. That is why there is a break in between obstetric services. They are nomads; moving together with cattle.”* [42 years old, KII participant, PHCU MCH coordinator, SNNPR]

### 3. Health service-related barrier

All participants discussed that shortage of supplies, poor infrastructure, and provider-related conditions were the main health service barriers to obstetrics service uptake.

**Poor supplies and infrastructure:** Most of the participants reported that a shortage of basic laboratory reagents and lack of equipment were major problems.

*“The lack of basic reagents and the availability of ultrasound services has forced us to send the mother to another health facility. This makes her ask about the need of going to a health facility and decide not to go to a health facility for a follow-up visit* [23 years old FGD participant, QIT Team, SNNPR]

On the other hand, the majority of key informants reported the absence of vital inputs to provide ANC services such as BP apparatus, fetoscope, adult weighing scale, examination coaches, laboratory reagents especially syphilis tests.

*“Instruments and material shortages are the other challenges of antenatal care service provision. Many health centers didn't have digital weight scales, examination coaches, ultrasound, screening tools, and other risk assessment tools and laboratory reagents, as a result, there are no baseline investigations for ANC* [43-year-old KII participant, WrHO MCH coordinator, Amhara region]

In the current study, infrastructure-related challenges were reported by the majority of respondents such as the absence of physical spaces such as rooms to provide reproductive health services.

*“…despite the narrowness of service delivery rooms, we are giving multiple services in one room. For instance, FP and ANC service is provided in one room, while EPI and SRH service in another room* [28 years KII participant, PHCU MCH focal, SNNPR]

Another KII participant strengthened that due to lack of wide room they discharge postnatal women despite the WHO standard.

*“There was a gap in the quality of PNC service provision. This was mainly due to a lack of infrastructure and a shortage of supplies. In most places we do have one narrow room for both delivery and postpartum care services because of this, health facilities keep mothers delivered in the health facility for only 6 hours.”* [KII, 36 years SNNPR RMNCH director]

The other barrier reported by the majority of informants was the closing or non-functionality of health posts.

*“There is a problem in the health extension program, where some health posts do not have health extension workers, while other health posts may not be open during service delivery hours. Closing of health posts is the main reason for the decreased performance of services…”* [ KII, 36 years SNNPR RMNCH coordinator]

**Provider related factors:** Unable to provide respectful and compassionate care was another challenge to obstetrics service provision.

*“From the health service provider perspective there are challenges like poor approach towards laboring mother and lack of respectful, compassionate, and caring health provider”* [27 years old FGD participant, Amhara region].

On the other hand, providers' competence was one of the issues raised by the majority of participants. One participant from the Sidama region reported graduates to have competence-related issues.

*“I told you there is a health professional competency problem. Those who graduated even from higher universities also have a large skill and knowledge gap. So it has also a great influence on the quality of service delivery. There is also a problem on the provision of respectful and compassionated service which have a great problem”* [36 years KII participant, MCH director, Sidama region]

**Monitoring and evaluation:** The majority of study participants reported that professionals who go to support HEWs have gaps in terms of knowledge and skill.

*“The health professional who went to support HEW for monitoring and support, in general, have a huge gap in knowledge and skill about the health extension program itself* [23-year-old KII, HEW, Oromia region].

Similarly, most participants also mentioned that poor linkage between PHCU and HP affects obstetrics service uptake which is mainly due to problems with access to road.

*“The linkage between the health center and health post and monitoring and evaluation was so poor. The health professional who went to support HEW for monitoring and support, in general, have huge knowledge and skill gap. In addition, there are remote HPs which didn't have road access so in those places cascading the monitoring and supervision process is so hard. Some health centers didn't have a motor bicycle and they go up to 12 km on foot to support one HP.”* [42 years KII participant, Sidama region]

### 4. Context related barrier

**Conflict/instability:** Most of the participants especially from the Amhara region and pastoral parts in the SNNP region stated that onset of conflict was their major barrier to obstetric service uptake. Home delivered mother also explained that conflict causes a huge barrier to accessing transportation and ambulance to get obstetrics services.

*“The main barrier for institutional delivery is lack of transport, poor client holding mechanism at a health facility, no contact address with the health care provider to get ambulance service; political instability makes the situation to be worst especially for the lack of ambulance because the ambulance is assigned for the civil war activity. On the other hand, most of the women did not know their date of labor, sudden onset of labor, and restriction of transportation due to the civil war.”* [26 years old home delivered mother, Amhara region].

**Topography and transportation:** Participants stated that distance, topography, transportation, and poor road access were major barriers in using facility-based delivery which in turn increases maternal mortality.

*“… because mothers come from a distant area and lack transportation services like ambulances and other options, we observed many mothers dying before reaching a health facility.”* [29 years KII participant, MCH case team leader, Oromia region,]

In agreement with the above other participants had raised concerns with regard to poor ambulance service availability exacerbating their participants further strengthened the above statement by saying:

*“…When labor starts early before the expected delivery date, we can't get timely ambulance service even if we call them. They say that the road is not safe for the ambulance. They also say that any pregnant mother should be at maternity waiting for home when her pregnancy reaches 8 months, otherwise they don't send ambulances”* [39 years FGD participant, WDT leader, Amhara region]

On the other hand, the majority of participants reported topography as one of the reasons for home delivery. HEW from the Sidama region reported topography as one of the reasons.

*“In our village, many mothers give birth at home because of topography or geographical conditions”* [29 years KII Participant, HEW, Sidama region]

Most of the other participants also mentioned there was a food shortage in the maternal waiting room due to budget shortages.

*“The other challenge was a shortage of food supplies in the maternity waiting room. The Woreda also allocates budget for maternity waiting room, but it is not adequate.”* [31 years male FGD participant, Quality member, SNNPR]

## Discussion

Barriers of maternal obstetrics health service utilization was explored by this study. Lack of awareness, unfavorable perception, lack of partner involvement, cultural barrier, shortage of supplies and poor infrastructure, provider related factor, poor monitoring and evaluation system, challenging topography and conflict were the main barriers of obstetric care service utilization which were presented using socio-ecological mode.

A pregnant women should have adequate knowledge about when and where to get obstetrics services which help her to start receiving the care early and increase maternal and child health (17). In line with this our study also explored that the main barrier to receive antenatal care is lack of awareness about when and where to start the care. Additionally, most of KII participants stated harmful cultural beliefs and practices were affecting the uptake of obstetric service which is mainly due to lack of awareness by the community in general. This finding also in line with the study done in other parts of Ethiopia (18,19).

In Ethiopia like other developing countries most of decision making is handled by males. They do have great role especially on obstetrics health service utilization(20). This study also identified that male involvement increases maternal health service utilization but in some parts of the country do not want to go with their wife to the health facility. Similarly, different studies also stated that partner involvement have great role in maternal health service utilization(21,22).

Participants also mentioned that shortage of supplies, poor infrastructure and closing of health posts (HPs) affect obstetrics service utilization. This finding also similar with other studies done in other parts of Ethiopia(18,23,24). This is due to the fact that lack of equipment, having poor infrastructure and closing of health facility are categorized under third delay, which is the major contributing factor for maternal mortality(25,26).

Respectful maternity care is very mandatory during providing maternal obstetrics care because disrespecting and abusive care prevents mother from receiving future utilization(27). In line with this our study also reviled that lack of respectful care is another barrier for service utilization. Providing abusive care might be liked to lack of trained staff which is in line with our study and this finding is also in line with other studies done in other parts of Ethiopia(28,29).

Monitoring and evaluation is a strategy that is very important to maintain quality of health service provision, which have effect on service uptake (30). Strong monitoring and evaluation system have effect on increasing the skill of the health care provider, motivate the health workers, increase resource allocation and strengthening implementing standard procedures. Study participants of this study also mentioned that poor monitoring and evaluation system is one of the barrier in obstetric service uptake (31).

Mother and children health was primarily affected during conflict period this is because it severely compromises health care delivery (32). It is known that in Ethiopia the conflict which was raised from the Tigray region was expanded to adjacent regions, Amhara and Afar, which causes significant effect on the health care delivery(33). Our study also demonstrated that due to conflict mothers could not get appropriate obstetric service even though they are in need.

This study also reviled that challenging distance or topography from the health facility is also barrier which prevents mother from acquiring maternal health service uptake(34). Participants of this study also mentioned that challenging topography and far distance from health facility increases home delivery. Even though the Ethiopian government launched a new innovating approach called maternal waiting home based on WHO recommendations to alleviate this problem, there is no sufficient food for pregnant women due to lack of budget(35). So, the problem is becoming a huge barrier.

In conclusion, different levels of barriers were identified through this study. Lack of awareness, problems with health system structure and process, conflict, and cultural and geographical conditions were major barriers in Ethiopia. Therefore, to alleviate these barriers the federal government should work strongly to manage raised conflicts in respective areas. The federal ministry of health in collaboration with other supporting governmental and non-governmental organization equip the health facilities and trains human resources. Regional health bureau, Zonal, and district level health offices should strongly work on increasing the community's awareness to decrease cultural beliefs and increasing accessibility of health facilities by strengthening maternal waiting rooms and through alternative ways. We also recommend implementing conflict resolution mechanisms and peace building are important for addressing better utilization of obstetric services.
